# The Dental Surgeon Defined

**Published:** 1844-03

**Authors:** C. T. Cushman

**Affiliations:** Columbus, Ga.


### ARTICLE VIII.

### *The Dental Surgeon Defined.* By C. T. CUSHMAN, Columbus, Ga.

IT has been held a popular opinion, that a "somerset" from the ordinary walks of life into the dental profession, is one of the easiest turns that can be made ; and with this idea, a great many are daily risking their necks by attempting the sudden transformation. There have been instances of individuals, with no qualifications by study, nor the teachings of experience, and even those who scarcely possessed sufficient common school education to enable them to write their own names, *graduating* at a *coup de main*, and, their "practice" in two or three years, has enabled them to *retire* into the luxuries of private life ! Such cases, or similar, have occurred all over the country, and tended to awaken the aspirations of their brother ostlers, corn-cutters, and mule-drovers, to follow in the same flight. But, the people have learned by experience—and they will have no other teacher—to beware of "land-sharks ;" they have, the most of them, by the aid of these *parvenu* professionals, "cut their eye-teeth," and learned to examine the pretensions of those who pretend to examine them. They have found that the bungling manipulations of those whose only physiological knowledge, and mechanical skill, consist in *intuitive perception*,* are of no benefit ; but, that often, as to be reasonably foreseen, they end in disfiguring forever those valuable appendages, so essential to the beauty and dignity of the countenance, and to the general health of the body—the teeth. They are thus placed beyond the power of art to restore, and no tears of regret can avail for such rashness.

* The reader has doubtless heard of "*Natural* Bone-Setters," and the like. I could refer to one of the same *genus* who professes to be a "Natural Dentist."

But it must not be inferred from the devastations of these individuals, from the fact that they have employed mineral acids, and barbarous usage upon these delicate organs—that the science of dental surgery is a chimera, and productive of no good—such a denunciation might be with no more injustice, applied to that of medicine, because of its flagrant abuses by unprincipled pretenders. It is a positive and demonstrative science; its remedies are not problematical in their effects, but determined, and visibly under control. As promulgated at the present day, especially by the American Society of Dental Surgeons, the only body of enlightened and scientific men in the history of the world, who have recognized it as a distinct and important one, of vital interest to the human family, it is founded upon everlasting truth.

Among all nations of the world are found the same natural diseases and defects of the dental organization, which enlightenment here has now learned to remedy, and the progress of art to overcome.

There are but few greater benefactors to humanity, than the learned and skilful dentist. For no person, perhaps, ever attained the age of twenty-five years, who had not need of his services. And, the amount of pain and of suffering, of mental anguish and lost comforts, of most people in after life, and in old age, resulting from diseases and loss of *the teeth*, is overwhelming to the contemplation. But there is a "Balm in Gilead." If he be seasonably employed, and frequently consulted, from time to time, he can secure all the requisites which combine to preserve through life these inestimable blessings, viz., cleanliness, regularity, health and beauty.

Again, there is no magic in a name, nor, in the promises of self-avarice. Are people too blind to see the fatalism of attempting to be, in the words of an English advertiser, "every one their own dentist"—by filling their decayed teeth with his paste, which one may do for himself, in a moment, and without pain!

The public should avoid being seduced by these "attractions," viz. "extraordinary cheapness," and operations "without pain." They may know that the one is attendant upon valueless services, and the other, a disregard for the patron's best interest. For it is evident, that the competent operator who is impressed with a due sense of his responsibitity, who employs sufficient time and perseverance, to do justice to his case; and, who uses a pure material, very costly in itself, must be liberally paid.

And where teeth are badly decayed, they are necessarily sensitive to the cutting instrument. "The wound must be probed to be healed," even so the decay must be faithfully cut out, to save the tooth.

An error of opinion is held by many physicians; that whereas they are conversant with the sciences taught in a Medical College, they are perfectly qualified by those studies, for the practice of Dental Surgery!

Medical works generally, are vague, and incorrect in their treatises upon diseases of the teeth, showing their ignorance of their true nature. And quite as defective are those on general anatomy and physiology, except, perhaps, some of late authorship. Physicians themselves, show that they have given but little, if any attention, to this branch of chirurgery. It is the remark of one of that faculty, who in early life turned his energies from the medical practice, entirely to that of dental surgery, and who by years of hard toil, made himself eminent also in its mechanical department—that the "physician's opinion on matters relating to the teeth, is no better than the parson's!"

This applied to those who are physicians *only*, will be found correct. They are, however, sometimes found operating on teeth, in the back part of their dingy shops, with an *unique* collection of clumsy "tools," (and a larger stock of self-conceit,) the chair, a common "windsor," and other fixtures in keeping. From these men we always see work that is of no utility; their scientific characters for other pursuits entirely fail to redeem it. He who aims at success, resting his faith upon abstract knowledge, would be as rational in expecting to manufacture a watch from his study of mechanics. A medical education is decidedly a valuable acquisition to the dentist; but particularly so is a *dental education* to *whosoever* would practice the art. Of what benefit can it be to the person who has carious teeth, to have them imperfectly excavated, and filled in a loose and unstable manner, though it be done by a medical man? By whomsoever, it is an *injury;* for *thus*, they are still pervious to the vitiated fluids of the mouth, and the stopping serves as a nucleus for their accumulation within the tooth, by which it must be more speedily decayed.

This most important and difficult operation—plugging teeth—is, in its perfection, the highest attainment of our art. But that cannot be reached, even by those who have the most learned *conceptions* of it, except by fatiguing experience, and the necessary means.

Nor is it given through *instinct* to the barber, the blacksmith, or the blockhead, who, too stupid in his vocation to obtain a livelihood, abandons it to honest men, barters his stock in trade for turnkeys and "gold leaf," and assumes his Sunday clothes, and the title of "Doctor."

Among the most important of these instrumentalities, to the want of which may be attributed the failure of professional and non-professional men, to arrive at that point of excellence, are the following:

Premising that it is folly to suppose a physician capable of doing justice to the practice of his profession, and, at the same time, to that of dental surgery—the latter in itself, is an ample field for a life of cultivating the best talents. There is no lack of eminent names to which we could refer as examples, for the truth of this.

The veteran PARMLY has even recommended that it be divided in practice, into two distinct branches—surgical and mechanical.

2dly. It is necessary to have a suitable *chair;* high enough to bring the patient's head up where one may *see* the state of the teeth. It is also needful to raise and lower the seat, the back, &c., as occasion may require. It should be *firm*, to give resistance to the pressure put upon gold in stopping teeth; and as that is often of the full strength of arm, to the end of making it perfectly solid, the chair must be unyielding to such force.

3dly. A good light is indispensable. Many operators literally *work in the dark,* and thereby work iniquity. For, without a *strong* light, they cannot see distinctly within a carious tooth, to know its true condition.

I have noticed where one advertiser is "prepared to operate in the evening, by gas-light!"

Large unshaded windows, fronting north, afford the clearest light. The chair should stand as near them as convenient, and the operator will find his labors much facilitated by placing a mirror in such side-position as to reflect the sun's rays directly into the mouth.

4thly. Delicate instruments are requisite; and of such various shapes, and peculiar niceties, that no person can make them properly, who does not know how to use them. The *cutler* manufactures from a given pattern, which, at best, is only imitation. As he is ignorant of their peculiar application, he cannot have an eye for their minute proportions. The skilful operator continually finds cases requiring a new pattern of instrument, or which suggest an improvement upon old ones, according to his idea of their use. Now, it is impossible to convey that idea to the cutler, by which he can make the precise instrument.

The dentist should not fail to supply himself with various sizes of steel wire, and learn to make his instruments, by forging and filing them down, tempering, polishing, &c. This should be one of his occupations during his leisure hours; and he will find a great portion of that time required to keep his instruments in *proper* order. For my part, if I had not others than which the cutlers can furnish, I should think it impossible to work. (This has reference particularly to those for excavating, and the smaller ones for plugging. The general excellence of the cutlery and surgical instruments manufactured in the northern cities is well known.)

5thly. A skilful *hand.* All other means will fail at last, if they be not directed by a disciplined, mechanical hand. It has been well remarked to the readers of this work, by high authority, that if it be not drilled to such delicate manipulations before the age of thirty years, it is afterwards incapable of that mastery which might have been obtained in early life. This fact is aptly illustrated by its application to musical instruments; it is invariably found, that after even a younger period, it is impossible to make up for neglect in youth, and to practise the hand into a free and elegant execution. The same is well known of the finer mechanical arts.

No mistake can be greater, than that the profession is one of ease and emolument, and that it requires but little preparatory qualification. He that justly claims the honor of belonging to it, is not content with any low or middle rank, but he must gain the very summit; and, to do this, requires, as has been fully shown, the longest life of unwearied diligence. The young dentist, on going forth into the world, has much to contend with. Though he be fitted for his responsible duties, at first the public rank him among those who have deceived them, and greet him with the contempt which they undoubtedly deserved. He, having too much self-respect to demean himself by low egotism, his success at the outset may not equal that of his *parvenu* contemporary, who thereupon takes occasion to republish his "extensive practice."

As the candidate for an honorable and lasting fame has in view to be *useful* to his patrons, he is never known to restore a decayed tooth in the astonishing space of twenty minutes; consequently, he does not surprise his auditors with extraordinary *day's work.*

His *entrée* is homogeneous to that of the young physician, than whom, perhaps, no candidate for public favor meets with more obstacles. The trials to which both are subjected, if known to those who would embark in such life for its pleasures, would reverse the ideal picture, and incline them to other pursuits.

In conclusion,—the young man possessing a liberal mind and education, a mechanical talent, and that spirit of enterprize which will direct all his energies to the improvement and enlightenment of himself and his calling, may feel assured that he can surmount all difficulties in this rugged path, which appears so falsely alluring to the thoughtlessness of youth, and so inviting to the avarice of the knave. And though for a while he meets with frowns from the world, yet his "good works" shall be perpetuated in the golden monuments he builds himself, proving him a benefactor to society, and an ornament to his profession.

